# Efficacy and Safety Outcomes of Bacteriophage Therapy for Resistant Bacterial Infections

**DOI:** 10.7759/cureus.108749

**Published:** 2026-05-12

**Authors:** Luke Cho, Hannah Chang, Evan Cho, Thiru Rajagopal

**Affiliations:** 1 College of Medicine, California Northstate University, Elk Grove, USA; 2 General Surgery, Mercy General Hospital, Sacramento, USA

**Keywords:** antibiotic-resistant bacterial infection, bacteriophage, management, outcomes, phage therapy, therapy

## Abstract

Phage therapy, the therapeutic use of bacteriophages for multi-drug resistant bacterial infections, is an understudied treatment approach that has been receiving increased interest in recent years. With multiple different treatment strategies (e.g., oral vs. intravenous (IV)) as well as varying types of bacteriophage combinations (phage cocktails) that are possible, the effectiveness of these phage cocktails on certain bacterial infections, as well as overall safety, is in question. This systematic review aims to analyze current evidence on the efficacy and safety outcomes of bacteriophage therapy for resistant bacterial infections. A systematic search of PubMed, Embase, and Scopus was conducted to investigate clinical studies following guidelines of the Preferred Reporting Items for Systematic Reviews and Meta-analyses (PRISMA). Included studies addressed the efficacy of phage cocktails in bacterial-resistant infections with a secondary focus on safety and adverse events. Two independent reviewers assessed randomized controlled studies using the Cochrane Risk-of-Bias 2 (ROB-2) tool and Risk of Bias in Non-randomized Studies of Interventions (ROBINS-I) tool. The three randomized controlled studies demonstrated low risk of bias, while the non-randomized controlled study showed a serious risk of bias. Patient outcomes and adverse events were narratively synthesized due to heterogeneity across study populations, pathogens, and treatment protocols. In total, only four studies met the scope of this review, which significantly limits the interpretation of the results. Two studies focused on *Pseudomonas aeruginosa *infections, while one study looked at *Mycobacterium spp.*, and another study examined *Helicobacter pylori* infections known to hold some antibiotic resistance*.* Phage administration ranged from topical/aerosol for the *P. aeruginosa *infections, IV/aerosol for *Mycobacterium spp*., and oral for *H. pylori.* The two studies on *P. aeruginosa *demonstrated statistically significant reductions in bacterial presence, while the other two studies did not demonstrate statistically significant results. Regarding safety, no study reported any statistically significant severe adverse events. Two trials provided data on less severe adverse events, which were not statistically significant compared to the comparison groups. The trends synthesized from this study are limited by disease and therapeutic heterogeneity, sample size, and different treatment durations. Phage therapy shows early promise for drug-resistant infections, particularly *P. aeruginosa*, with a favorable safety profile. However, due to therapeutic and disease heterogeneity, small sample sizes, and variable treatment durations, larger dedicated trials are needed.

## Introduction and background

Phage therapy, the therapeutic use of bacteriophages in treating bacterial infections, is a century-old modality that is currently experiencing a clinical resurgence. First developed in 1919 [1], phage therapy’s development was halted with the advent of antibiotics, but there has been growing interest in this modality as multidrug-resistant (MDR) bacteria increasingly threaten to usher in a “post-antibiotic era” [2]. Unlike traditional antibiotics, phage therapies utilize alternative routes of action that circumvent established resistance profiles while minimizing damage to the host microbiota and surrounding collateral tissue. Current therapeutic strategies range from “phage-cocktails" (a mixture of different bacteriophage types) that suppress potential bacterial resistance, to synergistic combinations with antibiotics to boost efficacy. Additionally, bioengineered phages utilizing Clustered Regularly Interspaced Short Palindromic Repeats (CRISPR) technology, a revolutionary gene-editing approach, have shown the potential to reverse bacterial resistance with increased precision [3]. Despite its proposed significance, clinical research on the effectiveness of phage therapy is still in its infancy. The PhagoBurn trial, initiated in 2010, while showing favorable outcomes in treating burn-wound *Pseudomonas aeruginosa*, was stopped prematurely due to limited efficacy [4]. There are additional ongoing trials investigating the use of phage therapy in animal models that have not yet transferred to human trials [5,6].

The mechanism of action behind bacteriophages follows the lytic and lysogenic viral life cycle, with the therapeutic application focusing on the lytic phase, which involves replicated viral progeny phages lysing the bacterial cell wall as they escape [7].

Given the recent interest in phage therapy and its treatment of MDR bacteria, there is limited research determining its safety and efficacy across various strains in human populations, despite its potential clinical benefit. Therefore, this systematic narrative review aims to synthesize current evidence regarding the outcomes of phage therapy in MDR infections and adverse effects associated with its use to further elucidate its clinical value.

## Review

Methods

Search Strategy and Selection Criteria

A search following guidelines established by the Preferred Reporting Items for Systematic Reviews and Meta-analyses (PRISMA) [8] was performed in three databases: PubMed, Embase, and Scopus. The query was performed utilizing the Boolean search phrase “((phage) OR (phage therapy) OR (phage cocktail) OR (bacteriophage [MeSH])) AND ((antibiotic resistance) OR (resistant) OR (MDR) OR (multi-drug resistant)) AND ((safety outcomes) OR (adverse events) OR (immunologic reactions))”. Search yielded results up until December 2025.

Study Selection

Covidence software (Covidence, Melbourne, Australia) was used to facilitate the screening of articles. Two independent reviewers reviewed studies for eligibility criteria from the initial database search using the specified inclusion and exclusion criteria. Any disputes were resolved through discussion and consensus. Studies were included if they evaluated bacteriophage therapy in human patients over 18 years of age diagnosed with drug-resistant bacterial infections. Included studies must have been peer-reviewed journal articles and reported clinical outcomes, including efficacy and adverse events. Exclusion criteria included case reports, review articles, conference abstracts, articles not in English, expert opinions, letters to editors, and studies in which phage therapy was not used to treat bacterial resistant infections in humans. This study was registered on the International Prospective Register of Systematic Reviews (PROSPERO; ID: CRD420261280056).

Data Extraction

Data were independently extracted using a standardized form on Microsoft Excel (Microsoft Corporation, Redmond, WA). Extracted variables included study characteristics, sample size, patient demographics, type of infection, causative organism, bacteriophage therapy type, route of administration, duration of therapy, patient outcomes, and reported adverse events.

Statistical Analysis

Given the heterogeneity of study design, patient population, and reported outcomes, a qualitative synthesis was performed. Study characteristics and reported outcomes were summarized. Where applicable, frequencies and proportions were reported for both patient outcomes and adverse events. A meta-analysis was not conducted due to the small number of studies that met the inclusion criteria, as well as heterogeneity between the studies.

*Qu* *ality Assessment and Risk of Bias*

Two independent reviewers utilized the Cochrane Risk-of-Bias 2 (ROB-2) tool [9] and the Risk of Bias in Non-randomized Studies of Interventions (ROBINS-I) tool [10] to assess the quality of included studies for randomized and nonrandomized controlled studies. This tool assesses bias across multiple domains, and each domain is categorized as having a “serious,” “some,” “moderate,” or “low” risk of bias.

Results

Literature Selection

**Figure 1:**
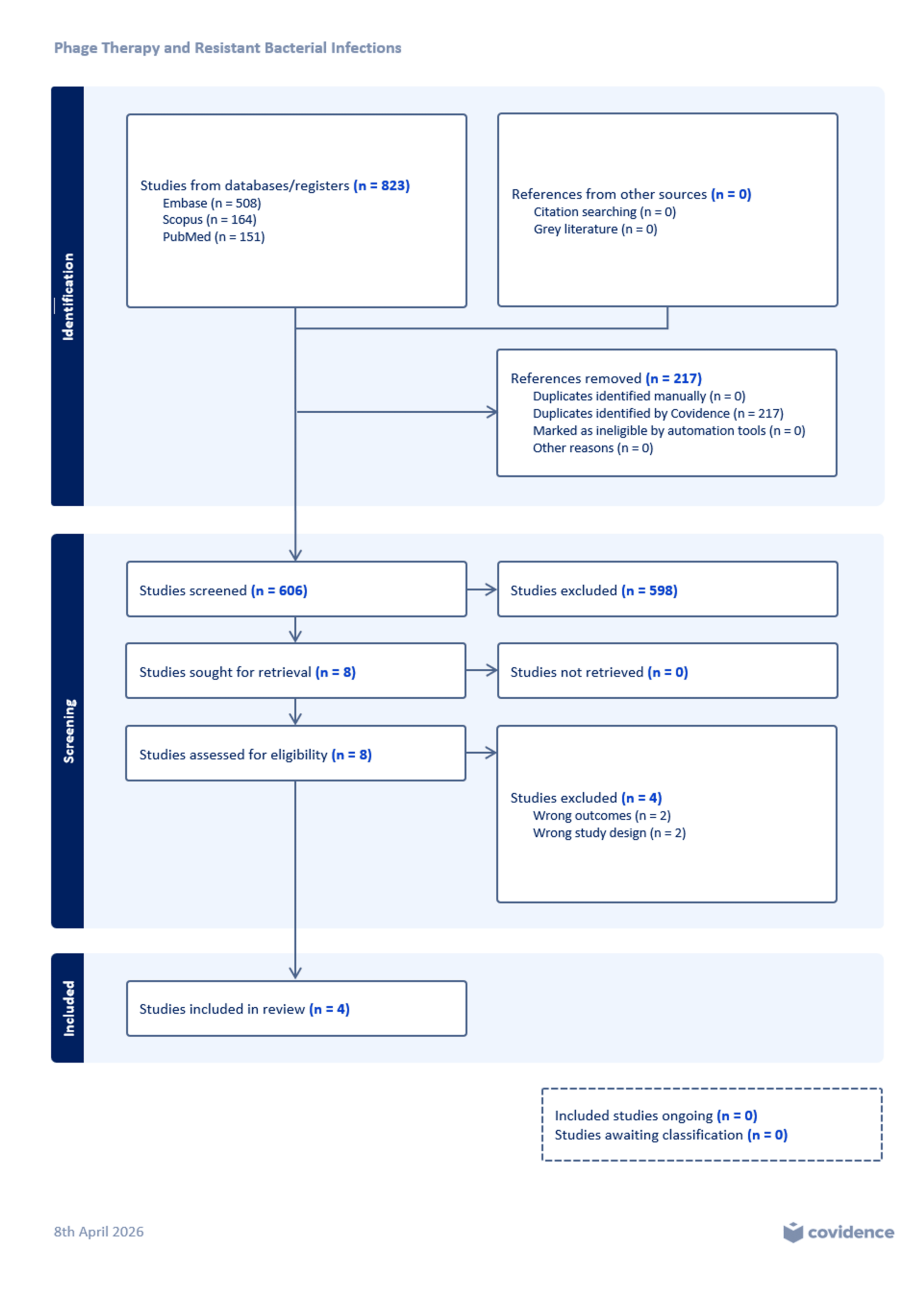
PRISMA flow diagram. PRISMA: Preferred Reporting Items for Systematic Reviews and Meta-analyses.

Our initial search using PubMed, Embase, and Scopus yielded 823 studies to screen. A total of 217 duplicate articles were removed, leaving 606 articles for title and abstract screening. Of these, 598 articles were excluded based on the inclusion and exclusion criteria, leaving eight articles for full-text screening. Of the eight articles, two had the wrong study design, and two reported wrong outcomes. Ultimately, four studies met all the inclusion criteria for this systematic review with no high risk of bias: Wright et al. [11], Dedrick et al. [12], Verma et al. [13], and Weiner et al. [14]. The PRISMA flow diagram (Figure 1) summarizes the study selection process.

Quality Assessment and Risk of Bias

The Cochrane ROB-2 and ROBINS-I tools were used to assess the risk of bias and the quality of included studies for randomized and nonrandomized controlled studies. Both tools assessed bias across multiple domains. Cochrane ROB-2 categorized each domain as having a "low," "some," or "high" risk of bias, while the ROBINS-I tool categorized each domain as having a “low," "moderate," "serious,” or "critical" risk of bias. All included studies demonstrated low risk of bias. The full quality assessment and risk of bias can be found in Table 1.

**Table 1:** Quality assessment and risk of bias. ROB-2: Risk-of-Bias 2; ROBINS: Risk of Bias in Non-randomized Studies of Interventions.

Cochrane ROB-2 for randomized controlled studies							
Study	Bias arising from randomization	Bias due to deviations from intended interventions	Bias due to missing data	Bias due to the measurement of the outcome	Bias in the selection of the reported result	Overall bias	-
Wright et al. [11]	Low	Low	Low	Some	Low	Low	-
Verma et al. [13]	Low	Low	Low	Low	Low	Low	-
Weiner et al. [14]	Low	Low	Low	Low	Low	Low	-
ROBINS for non-randomized control studies							
Study	Bias due to confounding	Bias in the selection of participants	Bias in the classification of interventions	Bias due to deviations in intended interventions	Bias due to missing data	Bias in the measurement of outcomes	Bias in the selection of reported results
Dedrick et al. [12]	Serious	Moderate	Low	Low	Low	Moderate	Low

Study Characteristics and Infection Profiles & Pathogens

Our results for this review yielded a small number of studies, highlighting the scarcity of clinical trials utilizing bacteriophages for the therapeutic use of MDR bacterial infections. Four studies met the inclusion criteria, of which two were randomized controlled trials (RCTs), one double-blind RCT, and one clinical observational study. Sample sizes ranged from nine to 50 participants, with a variation in the number of participants receiving the intervention. Across the studies, seven to 26 participants received the intervention. Wright et al. [11] evaluated *Pseudomonas aeruginosa* infections in otitis externa patients. Dedrick et al. [12] investigated pulmonary infections caused by *Mycobacterium spp*. Verma et al. [13] evaluated the treatment of *Helicobacter pylori* infection for dyspepsia. Finally, Weiner et al. [14] also evaluated *P. aeruginosa* in cystic fibrosis patients. Study characteristics and infection profiles are summarized in Table 2.

**Table 2:** Study characteristics and infection profiles & pathogens. RCT: randomized controlled trial; n: number of patients; n-treated: number of patients given a phage-based treatment.

Study	Wright et al. [11]	Dedrick et al. [12]	Verma et al. [13]	Weiner et al. [14]
Study characteristics and patient demographics				
Study type	RCT	Clinical observation	RCT	Double-blind RCT
n	24	20	50	9
n-treated	12	13	26	7
Infection profiles and pathogens				
Disease location	External ear (otitis externa)	Pulmonary infections	Gastrointestinal (dyspepsia)	Pulmonary (cystic fibrosis)
Pathogen	P. aeruginosa	Mycobacterium spp.	H. pylori	P. aeruginosa

Treatment Protocols

Treatment protocols varied across studies in treatment duration, route of administration, and concomitant use of antibiotics, as shown in Table 3. Median treatment duration varied between studies, ranging from seven days to nine months. In Wright et al. [11], patients were treated topically for a median of 42 days with no other concurrent antibiotic therapy. Dedrick et al. [12] had the longest median duration of treatment for six to nine months. Patients were treated using intravenous and aerosolized administration with concurrent use of isoniazid for some patients. In Verma et al. [13], patients received oral treatment with some in combination with standard medical therapy (SMT), including proton pump inhibitors, clarithromycin, and amoxicillin. Treatment was for a median duration of 14 days. In Weiner et al. [14], participants received a seven-day treatment course through aerosolized administration in combination with antibiotics.

**Table 3:** Treatment protocols. SMT: standard medical therapy.

Study	Wright et al. [11]	Dedrick et al. [12]	Verma et al. [13]	Weiner et al. [14]
Median treatment duration	42 days	6-9 months	14 days	7 days
Route	Topical	IV/aerosol	Oral	Nebulized
Concomitant antibiotics	N/A	Yes (Isoniazid) for some)	Yes (SMT triple therapy for some)	Yes (part of inclusion criteria)

Patient Outcomes

Due to heterogeneity between studies, patient outcomes could not be reported in one concise table, and results will be reported per individual study.

In Wright et al. [11], significant changes were reported in the treatment group receiving topical phage therapy compared to day 0 samples in colony-forming units (CFU), as shown in Table 4. Statistically significant decreases in CFU were reported specifically on days 21 (17.4% of day 0) and 42 (23.9% of day 0). In contrast, the placebo group reported no significant changes in CFU compared to day 0, with day 21 yielding 78.5% of day 0, and day 42 increasing to 108.9% of day 0. Interestingly, both treatment and placebo groups followed an initial decrease in CFU size on days seven and 21, followed by an increase in CFU on day 42. In terms of symptom resolution, this study utilized the visual analog scale (VAS) scoring system for both patients and physicians to determine changes in symptoms following treatment. The assessed variables for physicians included erythema/inflammation, ulceration/granulation/polyps, discharge type, and odor, while variables for patients were discomfort, itchiness, and wetness. In the treatment group, all but one patient improved, with three patients having an almost complete recovery (more than 80% reduction in VAS scores). In the placebo group, nine improved and three reported worsening symptoms, with no placebo patient demonstrating improvement above 80%. Both patient and physician groups showed statistically significant (P < 0.05) reductions in VAS scores from baseline for combined data from all clinic days, while the placebo group reported no statistically significant changes in both physician and patient VAS scores [11].

**Table 4:** Summary of Wright et al. Treatment of P. aeruginosa external ear infections with topical phage therapy results in colony-forming units (CFU). Values represent change from baseline in CFU counts. Within-group comparisons were performed using the Wilcoxon signed-rank test. Statistical significance is defined as P < 0.05. * Cumulative P-value for the placebo group: P = 0.835 (not significant).

Day	Treatment group (CFU)	Placebo (CFU) (P-value*)
0	9.3E + 09	6.5E + 09
7	5.3E + 09	9.2E + 09
21	1.6E + 09 (P = 0.009)	5.1E + 09
42	2.2E + 09 (P = 0.016)	7.1E + 09

In Dedrick et al. [12], studying IV and aerosol phage therapy response to *Mycobacterium spp.* infections, results were reported using a response scale (no response, inconclusive, partial/favorable response). The study reports the definitions of partial and favorable results as follows, “favorable response: mycobacterial smear and culture conversion to negative in at least 1 relevant specimen coupled with clinical and/or radiographic improvement or resolution of signs and symptoms of infections after at least 6-8 weeks of tx […] Partial response: either mycobacterial smear or culture conversion or clinical and radiographic improvement” [12].

Out of 13 patients, three reported favorable responses, two reported partial responses, and four each reported inconclusive and no response. In the partial response group, one patient demonstrated improvement of symptoms and forced expiratory volume in one second (FEV1), but sputum cultures remained positive. Despite demonstrating improved control of the mycobacterial infection, this patient ultimately passed away from superimposed bacterial and fungal coinfections unrelated to the study intervention.

In Verma et al. [13], the study’s primary endpoint was complete eradication of *H. pylori* via the addition of oral phage therapy to standard triple and quadruple antibiotic therapies. There was no statistically significant difference between the SMT and combo treatment with *H. pylori *eradication results of 66.7% and 69.2%, respectively (P = 1). In terms of symptom improvement, both groups showed improvement after two weeks of therapy, with a higher proportion of symptom-free results in the combo group across all domains. Despite this, results were not statistically significant, although the authors cite that the data suggest a trend toward better symptomatic relief with the addition of phage therapy adjuncts to standard therapy [13].

Finally, our last study, Weiner et al. [14], looked at changes in CFU in sputum samples from cystic fibrosis (CF) patients receiving nebulized phage therapy. While the study did not report individual responses, it demonstrated a significant reduction in *P. aeruginosa* CFUs in the patients receiving phage therapy compared to the control. The study highlights day four (1.9 log CFU per gram of sputum between groups, p = 0.035) and day 15 (2.7 log CFU per gram of sputum between groups, p = 0.029) with statistical significance defined as P < 0.05.

Adverse Events

In terms of adverse events, no study reported any severe adverse events. Two studies reported additional adverse events summarized in Table 5. The authors of Verma et al. [13] did not include statistical analyses of their adverse events, but in general reported a similar distribution of symptoms between both the SMT group and the SMT + phage group (combo). Notably, rates of headache and nausea were increased in the SMT group and not reported in the combo group. Additionally, the combo group reported a small percentage of patients developing hypothyroidism as an adverse effect of the phage treatment. Again, however, these results have not been given a significance value. In Wright et al. [11], no severe adverse events were reported between the phage and placebo groups. This study also did not report a statistical analysis of the adverse events. These findings are also summarized in Table 5.

**Table 5:** Adverse events of phage therapy as reported by Verma et al. and Wright et al. % = all percentages are expressed as the percentage of the number of volunteers in each treatment group (e.g., placebo, phage, etc.).

Adverse event	Headache	Nausea	Abdominal pain	Gastritis	Belching	Hypothyroidism	Ear pain	Thirst	Insomnia	Pharyngo-laryngeal pain	Rash
Verma et al. [13] (oral route of entry)											
Standard medical therapy (SMT)	4.17%	4.17%	50%	100%	95.83%	0%	-	-	-	-	-
Combo (phage + SMT)	0%	0%	46.15%	100%	96.15%	3.85%	-	-	-	-	-
Wright et al. [11] (topical route of entry)											
Placebo	25%	25%		-	-	-	17%	0%	0%	0%	0%
Phage	33%	8%		-	-	-	8%	8%	8%	8%	8%

In summary, microbiological reduction was demonstrated in both *P. aeruginosa* studies; clinical response was mixed in the observational *Mycobacterium spp*. study, and phage addition did not significantly improve eradication in the one *H. pylori* RCT.

Discussion

Efficacy of Phage Therapy

Based on the data collected in this study, several observations can be drawn. Because two of the studies, Wright et al. [11] and Weiner et al. [14], demonstrated statistically significant reductions in colonies of *P. aeruginosa *using phage therapy, it can be inferred that there is a potential benefit for this treatment for this specific bacterial species. Due to a lack of additional studies and other limiting factors (discussed below), it cannot be said with certainty whether phage therapy is effective for *P. aeruginosa*. Likewise, we cannot say with certainty that phage therapy is ineffective for *Mycobacterium spp.* and *H. pylori* specimens despite the insignificant findings of Dedrick et al. [12] and Verma et al. [13], respectively.

There are many animal studies detailing the effectiveness of phage therapy on other resistant bacterial infections, such as *Klebsiella pneumoniae* [15-17], *Acinetobacter baumannii* [18,19], and *Escherichia coli* [20,21]. One systematic review and meta-analysis of 124 preclinical animal studies demonstrated that phage therapy significantly improved animal survival across multiple infection types compared to placebo. Effectiveness was seen in systemic infections, skin infections, and pneumonia models [22].

Role of Combination Therapy

Three of the four studies detailed concomitant treatments that were given with phage therapy. In Dedrick et al. [12], a few patients with known tuberculosis infection were being treated with isoniazid. In Verma et al. [13], all patients received standard triple/quadruple therapy for *H. pylori* infections. Finally, all of the participants in Weiner et al. [14] received concomitant antibiotics along with their phage cocktails. Due to heterogeneity between studies, it is unclear whether there was a benefit to adjunct therapies in addition to the phage therapies.

Multiple animal studies have examined whether parallel treatment provides an additional benefit to phage therapy. Single phage vs. multiple phage (cocktail design) was explored in a few studies as well. One such paper identified that phages with complementary receptor targets prevent resistance emergence more effectively than single phages [15,23]. Additionally, another study found a synergistic effect with antibiotics that produced higher bacterial death rates than either treatment alone [16,24]. Further study into the synergistic effect of antibiotics with phages could be a worthwhile direction in the future.

Adverse Events

Among all four studies, none reported significant adverse events across all patients. Two studies, Verma et al. [13] and Wright et al. [11], demonstrated mild adverse effects (see Table 5) that were statistically insignificant compared to the SMT and placebo groups, respectively. While there is not enough data to say for certain that phage therapy has minimal adverse effects on human populations, the lack of significant adverse data points indicates a potential safety benefit to this therapy for antibiotic-resistant bacterial infections.

The literature also supports the safety of phage therapy among animal populations [22,24-27]. A paper in The Lancet conducted a comprehensive systematic review among 124 studies, finding that adverse events only occurred in 7% of phage-treated groups compared to 15% in controls [28]. The adverse events that did occur were not severe and typically resolved after treatment.

Limitations and Heterogeneity Between Studies

The following limitations should be considered when interpreting the findings of this systematic review. The number of available studies using bacteriophage therapies was few, along with the sample sizes being limited, which reduced the generalizability of the results. Time frames and measurement of reported outcomes varied between the studies, making direct comparison challenging. The safety profile could not be evaluated due to adverse events being reported by only two studies.

Additionally, significant heterogeneity was observed in the studies' design, outcomes, and interventions, which limited the ability to perform a meta-analysis. Despite the promising results of both efficacy and safety involving bacteriophage therapy, much research is still needed to improve our understanding of this therapeutic process. Within our clinical criteria, only four studies were found adequate to extract data from to create this review. More studies involving phage therapy for resistant bacterial infections in human patients are needed to support our suggested findings. Additionally, each study was very different from one another. As an example, although Wright et al. [11] and Weiner et al. [14] both studied phage therapy in *P. aeruginosa*, there are many differences between the studies. Wright et al. [11] administered phage therapy alone, whereas Weiner et al. [14] paired it with concurrent antibiotics. Wright et al. [11] provided phage therapy in a topical fashion, while Weiner et al. [14] used a nebulized route of entry. The other two studies also had different forms of medication delivery as well as preparations for their phage concoctions. All four studies had small sample sizes, the largest being Verma et al. [13] with 26 participants who underwent phage therapy. Finally, each study looked at different bacterial infections, making it difficult to determine which bacterial species phage therapy is best suited for.

Due to the limitations and differences between studies addressed above, substantial further investigation is required to verify the efficacy and safety of bacteriophage therapy on resistant bacterial infections. As such, there is also room to identify other applications of phage therapy as well.

Application of Bacteriophage Research With Animal Models

There are numerous animal models supporting the benefit of bacteriophage-coated surgical implants in preventing and treating implant-associated infections. Many studies suggest there is a large benefit for phage therapy for orthopedic implants [29,30]. One mouse study in 2025 demonstrated that triple-action titanium implants coated with bacteriophages and collagen provided a repellent lubricant layer against *P. aeruginosa*, with 100% survival rate compared to 30% with pathogen-repellent implants and 10% with untreated titanium [31]. Additionally, phages have also been studied for wound applications [32] and biomaterial delivery systems [33].

One notable absence in the literature is an application for anti-infective meshes for use in surgical procedures such as hernia repair. Although there are no specific studies regarding this, there is a recognized need to improve standard models for antibacterial meshes [34]. One study explains that phages have the ability to hydrolyze biofilm extracellular polymers, directly reducing the risk of device-associated infections [35]. Based on the animal studies regarding orthopedic implants and the clinical trials discussed in our study, exploring bacteriophage application to surgical mesh could be a worthwhile direction in the future.

Future Directions

Phage therapy has been studied in conjunction with CRISPR technology for several different applications. First, CRISPR-enhanced phage therapy has been studied as another method of implant infection prophylaxis [36,37]. As previously discussed, phage therapy has been shown in animal models to have a clear benefit in reducing bacterial-related infections of implants. However, with the addition of CRISPR, phages are able to provide specific vectors to target bacterial chromosomal DNA or antibiotic resistance genes, allowing for greater specificity, an advantage over conventional prophylactic antibiotics [38]. Second, phages are being combined with CRISPR to study targeted approaches to treating active bacterial infections, dubbed the phage-delivered resistance eradication with subsequent antibiotic treatment (PRESA) strategy. In mouse models, this therapeutic plan has been shown to decrease bacterial loads by over five to six logs and sustain efficacy without emergence of resistant mutants [39]. Finally, phages are being used to help streamline genome editing with CRISPR-Cas9 by providing effective methods of transporting, incorporating, and editing genes [40,41].

Finally, some institutions are developing so-called “phage banks” with the intention of creating a precision-medicine model for MDR infections. One such bank in UC San Diego, called The Center for Innovative Phage Applications and Therapeutics (IPATH), is now the home for many specific phage cocktails that can be shared and administered to patients needing specific, targeted therapy. One study on IPATH reported 785 requests for bacteriophage therapy between June 2018 and April 2020, with therapy recommended for 119 patients and administered to 17 of those patients [42].

## Conclusions

Phage therapy shows potential as an adjunct for treating MDR infections, particularly in combination with traditional antibiotics. The current evidence base, however, is limited by significant heterogeneity in administration routes, small sample sizes, and inconsistent safety reporting across patient populations. Phage therapies have strong potential benefits in the treatment of drug-resistant *P. aeruginosa *but require further studies to strengthen the case for their integration into clinical practice. Additionally, the eradication rate in *H. pylori* is promising, but large-scale RCTs are needed to establish definitive efficacy protocols and safety profiles before widespread clinical implementation. In summary, phage therapy has strong potential as an adjunctive treatment for MDR bacterial infections, but its clinical integration is currently limited by its lack of large-scale clinical trials across more strains detailing its safety and efficacy. Standardized, multicenter RCTs with larger patient populations and consistent outcome measures will be essential to establishing phage therapy as a viable, evidence-based treatment option in the era of rising antibiotic resistance.
